# Supplementary figures and images for: Altitudinal variation in reproductive investment among Gryllus campestris populations
Source: PLoS One. 2024 Oct 24;19(10):e0312048. doi: 10.1371/journal.pone.0312048 (PMC11500945; doi:10.1371/journal.pone.0312048)

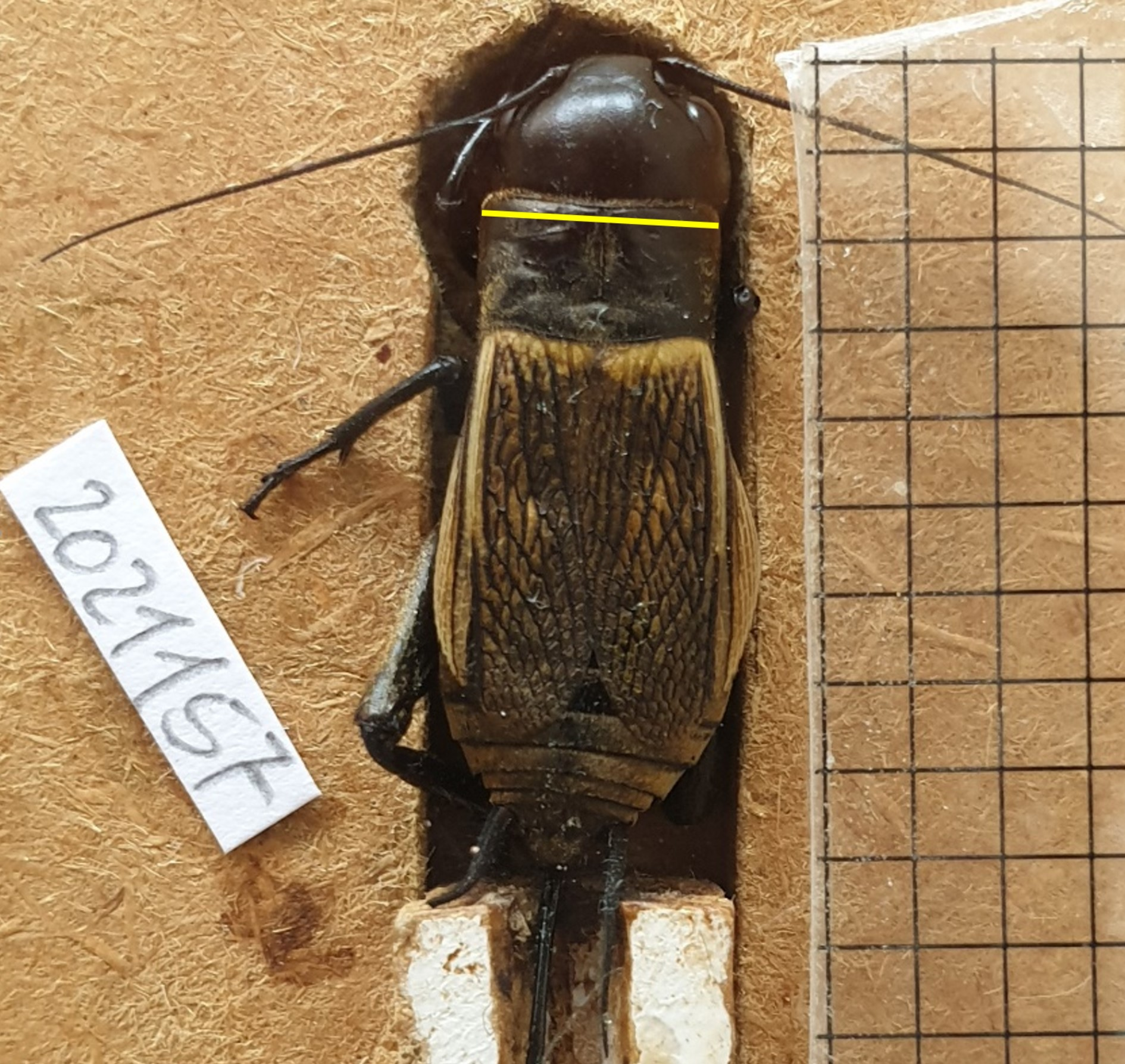

Supplement: S1 Fig — The yellow line represents the distance measured as thorax width. (TIF) [file pone.0312048.s001.tif]
