# Supplementary material for: Altitudinal variation in reproductive investment among Gryllus campestris populations
Source: PLoS One. 2024 Oct 24;19(10):e0312048. doi: 10.1371/journal.pone.0312048 (PMC11500945; doi:10.1371/journal.pone.0312048)
Supplement: Attachment — Submitted filename: PONE-D-24-00871_Response to Reviewers.docx [file pone.0312048.s002.docx]

PONE-D-24-00871

Altitudinal variation in reproductive investment among Gryllus campestris populations

PLOS ONE

Dear Dr. Rodríguez-Muñoz,

Thank you for submitting your manuscript to PLOS ONE. After careful consideration, we feel that it has merit but does not fully meet PLOS ONE’s publication criteria as it currently stands. Therefore, we invite you to submit a revised version of the manuscript that addresses the points raised during the review process.

**Both reviewers raised some concerns and provide several suggestions to improve the manuscript. One of the main issues relates to the experimental design (and the fact that all individuals were reared under the same environmental conditions) and the interpretation of the results. Please take into account the reviewer’s comments and carefully revise the manuscript before resubmitting.**

Please submit your revised manuscript by Apr 22 2024 11:59PM. If you will need more time than this to complete your revisions, please reply to this message or contact the journal office at [plosone@plos.org](mailto:plosone@plos.org). When you're ready to submit your revision, log on to <https://www.editorialmanager.com/pone/> and select the 'Submissions Needing Revision' folder to locate your manuscript file.

Please include the following items when submitting your revised manuscript:

- A rebuttal letter that responds to each point raised by the academic editor and reviewer(s). You should upload this letter as a separate file labeled 'Response to Reviewers'.
- A marked-up copy of your manuscript that highlights changes made to the original version. You should upload this as a separate file labeled 'Revised Manuscript with Track Changes'.
- An unmarked version of your revised paper without tracked changes. You should upload this as a separate file labeled 'Manuscript'.

If you would like to make changes to your financial disclosure, please include your updated statement in your cover letter. Guidelines for resubmitting your figure files are available below the reviewer comments at the end of this letter.

If applicable, we recommend that you deposit your laboratory protocols in protocols.io to enhance the reproducibility of your results. Protocols.io assigns your protocol its own identifier (DOI) so that it can be cited independently in the future. For instructions see: [https://journals.plos.org/plosone/s/submission-guidelines#loc-laboratory-protocols](https://eur03.safelinks.protection.outlook.com/?url=https%3A%2F%2Fjournals.plos.org%2Fplosone%2Fs%2Fsubmission-guidelines%23loc-laboratory-protocols&data=05%7C02%7Cr.rodriguez-munoz%40exeter.ac.uk%7Cb53ca7bca93a4ceb492108dc3fa0ec21%7C912a5d77fb984eeeaf321334d8f04a53%7C0%7C0%7C638455208072398275%7CUnknown%7CTWFpbGZsb3d8eyJWIjoiMC4wLjAwMDAiLCJQIjoiV2luMzIiLCJBTiI6Ik1haWwiLCJXVCI6Mn0%3D%7C0%7C%7C%7C&sdata=XbnyH6tArM67AwDCAgUXI8o6HbRLCYcHVV2UhVZppkU%3D&reserved=0). Additionally, PLOS ONE offers an option for publishing peer-reviewed Lab Protocol articles, which describe protocols hosted on protocols.io. Read more information on sharing protocols at [https://plos.org/protocols?utm_medium=editorial-email&utm_source=authorletters&utm_campaign=protocols](https://eur03.safelinks.protection.outlook.com/?url=https%3A%2F%2Fplos.org%2Fprotocols%3Futm_medium%3Deditorial-email%26utm_source%3Dauthorletters%26utm_campaign%3Dprotocols&data=05%7C02%7Cr.rodriguez-munoz%40exeter.ac.uk%7Cb53ca7bca93a4ceb492108dc3fa0ec21%7C912a5d77fb984eeeaf321334d8f04a53%7C0%7C0%7C638455208072406890%7CUnknown%7CTWFpbGZsb3d8eyJWIjoiMC4wLjAwMDAiLCJQIjoiV2luMzIiLCJBTiI6Ik1haWwiLCJXVCI6Mn0%3D%7C0%7C%7C%7C&sdata=OxZ4jwRHq2L7sn0FBdw9ln3ApSv6SOkLOIhojx3T0f8%3D&reserved=0).

We look forward to receiving your revised manuscript.

Kind regards,

Nicoletta Righini, PhD
Academic Editor
PLOS ONE

Journal Requirements:

*1. When submitting your revision, we need you to address these additional requirements.*

*Please ensure that your manuscript meets PLOS ONE's style requirements, including those for file naming. The PLOS ONE style templates can be found at* 
[*https://journals.plos.org/plosone/s/file?id=wjVg/PLOSOne_formatting_sample_main_body.pdf*](https://journals.plos.org/plosone/s/file?id=wjVg/PLOSOne_formatting_sample_main_body.pdf) *and* 
[*https://journals.plos.org/plosone/s/file?id=ba62/PLOSOne_formatting_sample_title_authors_affiliations.pdf*](https://journals.plos.org/plosone/s/file?id=ba62/PLOSOne_formatting_sample_title_authors_affiliations.pdf)

*2. In your Methods section, please provide additional information regarding the permits you obtained for the work. Please ensure you have included the full name of the authority that approved the field site access and, if no permits were required, a brief statement explaining why.*

**We have clarified this in the methods and included a reference in the acknowledgements.**

*3. Thank you for stating the following financial disclosure: 
NERC An individual-level approach to understanding responses to climate in wild ectotherms  (Climate & fitness): NE/V000772/1
 
Please state what role the funders took in the study.  If the funders had no role, please state: "The funders had no role in study design, data collection and analysis, decision to publish, or preparation of the manuscript." 
If this statement is not correct you must amend it as needed. 
Please include this amended Role of Funder statement in your cover letter; we will change the online submission form on your behalf.*

**The statement above is correct: “The funders had no role in study design, data collection and analysis, decision to publish, or preparation of the manuscript”**

*4. When completing the data availability statement of the submission form, you indicated that you will make your data available on acceptance. We strongly recommend all authors decide on a data sharing plan before acceptance, as the process can be lengthy and hold up publication timelines. Please note that, though access restrictions are acceptable now, your entire data will need to be made freely accessible if your manuscript is accepted for publication. This policy applies to all data except where public deposition would breach compliance with the protocol approved by your research ethics board. If you are unable to adhere to our open data policy, please kindly revise your statement to explain your reasoning and we will seek the editor's input on an exemption. Please be assured that, once you have provided your new statement, the assessment of your exemption will not hold up the peer review process.*

**We will make the data available on acceptance by storing it in the data repository of the Universidad de Oviedo (**[**https://digibuo.uniovi.es/dspace/page/repository?locale-attribute=en**](https://digibuo.uniovi.es/dspace/page/repository?locale-attribute=en)**). Uploading the data is very easy and fast, so we don’t expect this to cause any delay in the publication timeline.**

*5. PLOS requires an ORCID iD for the corresponding author in Editorial Manager on papers submitted after December 6th, 2016. Please ensure that you have an ORCID iD and that it is validated in Editorial Manager. To do this, go to ‘Update my Information’ (in the upper left-hand corner of the main menu), and click on the Fetch/Validate link next to the ORCID field. This will take you to the ORCID site and allow you to create a new iD or authenticate a pre-existing iD in Editorial Manager. Please see the following video for instructions on linking an ORCID iD to your Editorial Manager account:* [*https://www.youtube.com/watch?v=_xcclfuvtxQ*](https://www.youtube.com/watch?v=_xcclfuvtxQ)

**We have included ORCID iD for the corresponding author (0000-0002-6067-845X).**

*6. Please include captions for your Supporting Information files at the end of your manuscript, and update any in-text citations to match accordingly. Please see our Supporting Information guidelines for more information:* [*http://journals.plos.org/plosone/s/supporting-information*](http://journals.plos.org/plosone/s/supporting-information)*.*

**We have included the captions for the SI file in the last line of the manuscript (before the references).**

*[Note: HTML markup is below. Please do not edit.]

Reviewers' comments:

Reviewer's Responses to Questions*

*Comments to the Author

1. Is the manuscript technically sound, and do the data support the conclusions?

The manuscript must describe a technically sound piece of scientific research with data that supports the conclusions. Experiments must have been conducted rigorously, with appropriate controls, replication, and sample sizes. The conclusions must be drawn appropriately based on the data presented.*

*Reviewer #1: Partly*

*Reviewer #2: No*

*2. Has the statistical analysis been performed appropriately and rigorously?*

*Reviewer #1: Yes*

*Reviewer #2: Yes*

*3. Have the authors made all data underlying the findings in their manuscript fully available?

The* [*PLOS Data policy*](https://eur03.safelinks.protection.outlook.com/?url=http%3A%2F%2Fwww.plosone.org%2Fstatic%2Fpolicies.action%23sharing&data=05%7C02%7Cr.rodriguez-munoz%40exeter.ac.uk%7Cb53ca7bca93a4ceb492108dc3fa0ec21%7C912a5d77fb984eeeaf321334d8f04a53%7C0%7C0%7C638455208072411818%7CUnknown%7CTWFpbGZsb3d8eyJWIjoiMC4wLjAwMDAiLCJQIjoiV2luMzIiLCJBTiI6Ik1haWwiLCJXVCI6Mn0%3D%7C0%7C%7C%7C&sdata=5eHGsCJVh6Wai7Oa2rpnF3YWNIXQCRwLGnslOd616WM%3D&reserved=0) *requires authors to make all data underlying the findings described in their manuscript fully available without restriction, with rare exception (please refer to the Data Availability Statement in the manuscript PDF file). The data should be provided as part of the manuscript or its supporting information, or deposited to a public repository. For example, in addition to summary statistics, the data points behind means, medians and variance measures should be available. If there are restrictions on publicly sharing data—e.g. participant privacy or use of data from a third party—those must be specified.*

*Reviewer #1: No*

*Reviewer #2: No*

*4. Is the manuscript presented in an intelligible fashion and written in standard English?

PLOS ONE does not copyedit accepted manuscripts, so the language in submitted articles must be clear, correct, and unambiguous. Any typographical or grammatical errors should be corrected at revision, so please note any specific errors here.*

*Reviewer #1: Yes*

*Reviewer #2: Yes*

*5. Review Comments to the Author

Please use the space provided to explain your answers to the questions above. You may also include additional comments for the author, including concerns about dual publication, research ethics, or publication ethics. (Please upload your review as an attachment if it exceeds 20,000 characters)*

***Reviewer #1:*** *The submitted manuscript describes an experiment examining local adaptation in field cricket life history traits across an elevational gradient. The study collected adult and late-stage juveniles from five low-elevation and five high-elevation populations that were each separated by geographic barriers. Mature crickets were kept under common laboratory conditions. After mating, the study measured female body size, egg size, and egg laying rate. The study tested for elevational differences in female body size, along with the relationship between female body size and egg traits to test if reproductive investment strategies have evolved across elevations. The results are interpreted in the context of insect responses to climate change. Overall, the study is straightforward, and most of the methods are clearly articulated. The statistical analyses are elegant and justified. I have a few comments about limitations in the experimental design and interpretation of the results that I believe should be addressed in the manuscript before publication. However, I am generally supportive of the manuscript, and I believe a revision could meet the criteria for publication in the journal.*

*Comments:
1) The manuscript finds that phenotypes do not differ between low and high elevation populations. However, because crickets from each population were not reared under common or reciprocally transplanted conditions, the study cannot elucidate whether this lack of a phenotypic difference is due to (A) a lack of evolutionary change, (B) a lack of phenotypic plasticity, or (C) countergradient selection. The manuscript mentions in the introduction (line 71) that phenotypes could differ among environments due to evolved and/or plastic responses. However, the conclusions and abstract should clearly state that this study does not reveal the source of phenotypic similarities between elevations.*

**We have added a brief comment in the abstract and the discussion to make clear that our study design cannot identify the sources of variation, but just to assess the potential existence of differences between the two environments.**

*2) Similarly, there is well-established and taxonomically pervasive evidence that insect life histories often evolve and/or show phenotypic plasticity in response to environmental factors correlated with elevation (temperature, hydration, oxygen), making the study’s findings surprising. I believe many researchers would hypothesize that countergradient selection underlies the phenotypic similarities observed among these cricket populations. The Introduction and Discussion would be greatly improved by discussing this hypothesis. Conover & Schultz 1995 TREE (doi: 10.1016/S0169-5347(00)89081-3) would be a helpful reference for this discussion. A main goal of the manuscript is to interpret the results in the context of climate change responses. The patterns of evolution and/or plasticity that underly the phenotypic similarities observed here are important for determining how egg size strategies will respond to climate change in different populations, and this should be addressed in the discussion. For instance, if phenotypic similarities are due to a lack of evolutionary change and a lack of developmental plasticity across elevations, then the phenotypes will likely be robust in the face of climate change. However, if phenotypic similarities stem from countergradient selection, then climate change could still alter cricket life history traits.*

**We agree about the need to include this in the discussion, and we have done so. As both reviewers point out, this is a very simple study that does not allow to separate the components behind what we have found, and this is particularly relevant in there is countergradient variation. However, during their breeding season, crickets have the possibility to control their body temperature using behavioural thermoregulation whenever solar radiation is available (Gardner et al. 2024), and there seems to be no or very limited difference in thermal behaviour among adult crickets coming from different altitudes (Rodríguez-Muñoz et al. 2023). This suggests that the potential effect of the temperature experienced during the short time spent in the lab, as the cause of the observed similarities between altitudes could be small. We have added now all these questions to the discussion.**

*3) The study focuses on elevational differences in temperature when interpreting the results. Populations at different elevations experience differences in a number of environmental factors, including hydration and oxygen availability. The introduction and discussion would benefit from a more balanced discussion of these differences beyond temperature. Furthermore, more details regarding how laboratory conditions compared to environmental conditions in the low and high elevation populations should be included in the main text. Is 25C more representative of low or high elevation populations, or is it in-between?*

**We needed the lab period to mate the crickets and get eggs from the females. We used an ambient temperature that we think is close to the species optimum for both activities, which we think is probably not very different between both altitudes (see comment above), and in order to stimulate mating and egg laying. Regarding hydration, crickets had water ad libitum during their lab period, and water and food are also fully available in their sites of origin during the breeding season, so we don’t think this would be an issue. Differences in oxygen concentration do exist between altitudes, and the laboratory is located close to the central low altitude population (Figure 1, L03). We are not aware of studies showing an effect of oxygen concentration on egg size in this context, but we cannot rule it out. We have included some comments on this in the discussion.**

*4) The manuscript should provide more information about how many females from each population were known to have mated in the field (for example, in Table 1). It appears that low elevation populations mature earlier, and therefore are more likely to have females that mated in the field. If females mated multiple times in the field, then that could generate elevation differences (or similarities) in egg fertilization/laying. The manuscript would therefore be greatly improved by including supplemental analyses that remove data from females known to have mated in the field.*

**With the only exception of L01, where we caught them later, we collected all the crickets by the start of the reproductive season, which happens usually over the second half of April. This is supported by the presence of nymphs among the crickets caught in at three of the low altitude populations, and in all of the high altitude ones (see Table 1 in previous version). This means that mating activity should have been not very intense by then. However, part of the females did certainly mate before being collected. Indeed, there were 15 females that refused to mate in the lab but laid viable eggs, including 16% and 25% of the high and low altitude individuals, respectively. This shows that they mated in the wild before being moved into the lab. Apart from those, mating status could only be established by dissection or genotyping, but both things are not compatible or feasible within this study. Thus, there might be some other previously mated females at both altitudes in our sample, but we cannot detect them because they mated in the lab, and that allows them to lay eggs regardless of their previous mating status. Although there is a large difference in sample size, we used the same analytical approach described in the methods, to compare those 15 females that we know mated in the field, with the rest of the females in our sample. We found no differences in egg mass (*F_1,54.3_* =0.091; *P* = 0.764) or laying rate (*F_1,54.3_* =1.284; *P* = 0.263). However, we cannot totally rule out the existence of small differences in number of matings between altitudes that could have had some effect on the measured reproductive effort. We have made some changes and added some comments in the discussion about this.**

*5) The effect estimates in Table 3 seem very high, indicating that the data may not be standardized relative to the mean and standard deviation for these analyses. If not already done, then standardizing the data in this way is more appropriate for evaluating effect size (see Schielzeth 2010, Methods Ecology & Evolution, doi: 10.1111/j.2041-210x.2010.00012.x). This procedure should not change the reported P values.*

**We agree that standardizing the data is important in this type of design when there is a covariate (thorax width), as the intercepts are meaningless if there were an interaction between altitudes (although we did not find it). We have run again the analyses of egg mass and laying rate after stardardizing thorax width, and updated tables 2 and 3 accordingly. As explained by the reviewer, P-values did not change for the effect of the covariate, although they did for the rest. This is normal in mixed models using the Satterthwaite method for unequal sample sizes.**

*6) It is not clear why the sample sizes for populations L02 and L03 are not available if these populations were included in the analyses*.

**We realize that Table 1 was confusing. It showed the number of individuals we collected at each site for each sex and developmental stage, and not the actual numbers we included in the experiment. We included those numbers to provide some information about the phenology (proportion of nymphs vs. adults) of each population by the time of capture. We agree that the number of crickets included in the analyses is actually the most relevant information, so we have updated the table to remove the information in crickets collected and show the actual samples size per population for each of the three variables.**

*7) Following the journal’s guidelines for data availability, the authors should deposit relevant data in a public data repository or provide the data in the manuscript, rather than have data available upon request. Figure 2 could also be improved by adding the data points behind the means and standard errors.*

**If the manuscript is accepted for publication, we will deposit the dataset in the data repository of the Universidad de Oviedo (**[**https://digibuo.uniovi.es/dspace/page/repository?locale-attribute=en**](https://digibuo.uniovi.es/dspace/page/repository?locale-attribute=en)**). We have added the data points to figure 2.**

**Reviewer #2:** Rev PONE D-24-871

*l38 …both traits were analysed controlling for female size.*

**Changed**

*180-81 … such as temperature…*

**Changed**

*l88 delete “faithfully”*

**Changed**

*l100-101 it is not clear until you read the methods why “laying rate”, I suggest to simply say “egg number” at this point*

**Changed**

*l101 We expected…*

**Changed**

*l106 …would be smaller and would lay…*

**Changed**

*l107-109 Delete last sentence in intro, this can be mentioned in the discussion.*

**Removed**

*l116 the abstract said under 170 masl and here 200 m, chose one for consistency.*

**170 is the right number. We have amended this.**

*l126 why are number of captured crickets “not available” for two sites? Report the numbers that were used for analyses. Even if more were captured, they are irrelevant; if not explain why?. Why are there two sites labeled as L05? If they represent a single site then merge them in only one line in the table. “XYZ” column tittles for coordinates and altitude seem too cryptic, label with more details and include units (UTM coordinates, m asl). Could you provide some columns summarizing relevant climate variables for each site such as mean temperature, average date of first and last below 0ºC temperature in the year, average rainfall, degree days or anything you think could relate to reproductive performance. It would be good to see if altitude differences really represent a sizable climate variation, and these data are easy to get nowadays for almost any point in the world, certainly for Northern Spain*

**We agree with these comments. See reply to question 6 from reviewer 1 regarding number of crickets. We have relabelled the columns showing the coordinates and altitude, and explained in the caption what type of information they show. Population L05 was sampled at two different locations less than 5 km apart. To avoid confusion, we have now included the coordinates of the central point between both in a single row. Regarding climatic data, as far as we know, there are no weather stations at or near most of the locations where the different populations were originated. Although there is the option to use available climatic models to estimate values for each location, we have opted for including data from two weather stations that we’ve had in operation over the last three years, one of them at the highest altitude collection site (1,300 masl), and the other very near the central low altitude site (70 masl). We have included mean annual temperature and mean number of days with maximum temperature below 10 ºC, just to give an idea about the differences between the two altitude environments included in the study, in two variables that we think are particularly relevant for an insect.**

*1122 Review map, after converting coordinates for L1 and H10 and projecting on Google Earth, the coastline does not match and the scale that should represent 20 km is actually 33 km. See attached png file.*

**Indeed, there is a mismatch in the relative size of the scale and the map that we have now corrected. We thank the reviewer for having spotted this weird mistake.** *l132 space between 2 and l?*

**We have corrected this and all other similar mistakes**
 *l137-138 Where was this building geographically compared to the collecting sites? Comparing the climate regime of the lab to the collecting sites may be useful for interpretation of results as this is where females ended reproductive maturity and spent most of their egg laying period, thus reproductive “choices” may have been greatly influenced by these conditions, and since they were all in the same conditions, this may a feasible explanation for the results of the study.*

**See comment to question 3 of reviewer 1 above. The lab is located at low altitude, near collection site L03 (see Figure 1). As both reviewers have pointed out, this study does not allow to separate genetic from environmental sources of variation, and also, we cannot assess how important is the effect of the environment by the time of reproduction in relation to the expression of the analysed variables. Most of the females were already adults and reproductively mature when they arrived to the lab, so they spent most of their life in the conditions of their source environment. Although we don’t think the differences between altitudes by the time of reproduction are very relevant (see comment to question 3 of reviewer 1 above), we cannot discard possible effects on egg size and fecundity. We have included this in the discussion.**

*162 “A maximum” can only have one value, 3 or 5? If the max value differs between samples, explain why.*

**There are two different reasons why we did not try to mate all females 5 times before discarding them. First, some populations had more females available than others, and for those we decided to try new females rather than keep trying the same female more times. Second, sometimes we could not do more mating trials in due time due to logistic reasons. Nevertheless, 6 females mated at the fourth or fifth trial. We do not believe this is likely to have had an effect on the analysed traits, but we could remove them if the reviewer thinks including them would be problematic.**

*l169 why about 20 eggs and not simply exactly 20 eggs each time. Were these eggs taken randomly throughout the period? Could egg size differ at different periods of the reproductive period thus?*

**As a matter of fact, we collected exactly 20 eggs per female, but in some samples we found 1-2 broken or clearly deteriorated eggs by the time when the egg mass was about to be measured, so we reported it like ⁓ 20 eggs. Because we finally estimated egg size by weighing three samples of 5 eggs for each female (as described in the methods), the number initially stored in the sample is actually not relevant. We have now clarified this in the methods. We collected the sample within the first 13 days after laying started, and only when the number of eggs in the batch was more than 19. Mean number of days between first laying and sample collection was 3.1 and 4.4 for high and low altitude, respectively. Because most of the females in the study were already adult when we caught them, we do not know exactly how old they were. Also, we do not know if egg size could vary with female age. This means that there is a possibility that female age by the time when we collected the sample of eggs could be adding noise to the data.**

*l202 if female size were perfectly correlated with egg mass, thorax width would increase as a linear function but egg mass as a cubic function, so it is better to use cubic thorax width as a covariate and in all analyses involving egg mass at least.*

**Not sure about why the reviewer is suggesting this change. We have tested it and using cubic thorax width does not improve the fitting.**

*l217 was laying rate normally distributed? Rates frequently are not. Only if it was mean and a standard deviation values would be meaningful.
Otherwise use medians and quartiles.*

**The distribution is not bimodal or clearly biased, so we think it is appropriated using mean and standard deviation.**

*l219 use “egg mass” instead of “egg size” since the former is the actual operational value you measured*

**Changed** *l230 remove the word “Low” from the table as you are presenting results for the “Altitude” variable*

**Not sure about what the reviewer means in here. The word “Low” in the table is required to show that the altitude reference level in the analysis is “High”.** *l239-334 I provide general comments for the discussion. No specific comments on the text are provided as I did for the previous sections since I suggest that the whole discussion should be rewritten due to two main reasons:

The design and interpretation of results assumes that any difference between high and low populations would be maintained outside their natural climate environment, in other words that there would be no plasticity in reproductive output even though phenotypic plasticity is mentioned in the introduction, thus it should be considered in the discussion of the results as well. Since all individuals were reared under the same environmental conditions after being collected and throughout the last part of their developmental period in many cases, and most of their reproductive period, they had plenty of time to “adjust” to the experimental rearing conditions, possibly explaining the lack of significant effects of altitude. It would be ideal to also measure reproductive output if maintained in their original condition, and compared to those that were placed under the new experimental conditions, or have actually crossed conditions (high altitude moved to low altitude conditions and vice versa plus the appropriate control groups), but the present experimental design opens the possibility of plastic adjustment to experimental conditions but offers no way to assess if indeed it happened.
Non-significant results can not be treated with the same logic of significant results, in other words, lack of significant differences can not be directly translated into no biological difference since there is always the possibility of low statistical power, among other reasons. There is extensive literature discussing this topic and the authors should refer to it, just to give 2 examples I offer these references, buth there are many others:*

[*https://www.ncbi.nlm.nih.gov/pmc/articles/PMC4114196/*](https://www.ncbi.nlm.nih.gov/pmc/articles/PMC4114196/)
[*https://online.ucpress.edu/collabra/article/3/1/9/112371/Too-Good-to-be-False-Nonsignificant-Results*](https://online.ucpress.edu/collabra/article/3/1/9/112371/Too-Good-to-be-False-Nonsignificant-Results) *Most of the discussion is built on the assumption that these results are solid evidence of no biological difference between high and low populations, when they are only statistically non significant differences, which is VERY different.*

**We totally agree with the reviewer that there is no way our study can arrive to the conclusion that we found solid evidence of no biological difference. It wasn’t our intention at all. We have re-written the discussion in relation to the suggestions in here and in the comments above.**

6. PLOS authors have the option to publish the peer review history of their article ([what does this mean?](https://eur03.safelinks.protection.outlook.com/?url=https%3A%2F%2Fjournals.plos.org%2Fplosone%2Fs%2Feditorial-and-peer-review-process%23loc-peer-review-history&data=05%7C02%7Cr.rodriguez-munoz%40exeter.ac.uk%7Cb53ca7bca93a4ceb492108dc3fa0ec21%7C912a5d77fb984eeeaf321334d8f04a53%7C0%7C0%7C638455208072416321%7CUnknown%7CTWFpbGZsb3d8eyJWIjoiMC4wLjAwMDAiLCJQIjoiV2luMzIiLCJBTiI6Ik1haWwiLCJXVCI6Mn0%3D%7C0%7C%7C%7C&sdata=kv7OtplOpB%2FLMp2YSP7HJ%2F5BCci979ERTbx3JNccJo8%3D&reserved=0)). If published, this will include your full peer review and any attached files.


If you choose “no”, your identity will remain anonymous but your review may still be made public.


**Do you want your identity to be public for this peer review?** For information about this choice, including consent withdrawal, please see our [Privacy Policy](https://eur03.safelinks.protection.outlook.com/?url=https%3A%2F%2Fwww.plos.org%2Fprivacy-policy&data=05%7C02%7Cr.rodriguez-munoz%40exeter.ac.uk%7Cb53ca7bca93a4ceb492108dc3fa0ec21%7C912a5d77fb984eeeaf321334d8f04a53%7C0%7C0%7C638455208072421284%7CUnknown%7CTWFpbGZsb3d8eyJWIjoiMC4wLjAwMDAiLCJQIjoiV2luMzIiLCJBTiI6Ik1haWwiLCJXVCI6Mn0%3D%7C0%7C%7C%7C&sdata=4Hs8AvhCxfWk78lgqIi8dHDytVdizhKkD9g50BfEkhM%3D&reserved=0).

Reviewer #1: No

Reviewer #2: **Yes:** Rogelio Macías-Ordóñez

**REFERENCES**

**Gardner, A. S., I. M. Maclean, R. Rodríguez‐Muñoz, P. E. Hopwood, K. Mills, R. Wotherspoon, and T. Tregenza. 2024. The relationship between the body and air temperature in a terrestrial ectotherm. Ecology and Evolution 14:e11019.**

**Rodríguez-Muñoz, R., P. Hopwood, J. J. Boonekamp, S. A. Edwards, T. W. Price, J. Rees, and T. Tregenza. 2023. Local adaptation does not constrain the expression of behaviour in translocated wild crickets. Anim. Behav. 200:15-23.**
