# Supplementary material for: Altitudinal variation in reproductive investment among Gryllus campestris populations
Source: PLoS One. 2024 Oct 24;19(10):e0312048. doi: 10.1371/journal.pone.0312048 (PMC11500945; doi:10.1371/journal.pone.0312048)
Supplement: Attachment — Submitted filename: PONE-D-24-00871R1_Response to Reviewers.docx [file pone.0312048.s003.docx]

*PONE-D-24-00871R1*

*Altitudinal variation in reproductive investment among Gryllus campestris populations*

*PLOS ONE*

*Dear Dr. Rodríguez-Muñoz,*

*Thank you for submitting your manuscript to PLOS ONE. After careful consideration, we feel that it has merit but does not fully meet PLOS ONE’s publication criteria as it currently stands. Therefore, we invite you to submit a revised version of the manuscript that addresses the points raised during the review process.*

*I agree with Reviewer 1 in that the ms has greatly improved and the authors responded adequately to the reviewers' queries. Reviewer 1 points out a couple of minor issues; once these are addressed, the ms can be accepted for publication.*

*Please submit your revised manuscript by Sep 16 2024 11:59PM. If you will need more time than this to complete your revisions, please reply to this message or contact the journal office*

*at plosone@plos.org. When you're ready to submit your revision, log on to https://www.editorialmanager.com/pone/ and select the 'Submissions Needing Revision' folder to locate your manuscript file.*

*Please include the following items when submitting your revised manuscript:*

- *A rebuttal letter that responds to each point raised by the academic editor and reviewer(s). You should upload this letter as a separate file labeled 'Response to Reviewers'.*
- *A marked-up copy of your manuscript that highlights changes made to the original version. You should upload this as a separate file labeled 'Revised Manuscript with Track Changes'.*
- *An unmarked version of your revised paper without tracked changes. You should upload this as a separate file labeled 'Manuscript'.*

*If you would like to make changes to your financial disclosure, please include your updated*

*statement in your cover letter. Guidelines for resubmitting your figure files are available below the reviewer comments at the end of this letter.*

*If applicable, we recommend that you deposit your laboratory protocols in protocols.io to enhance the reproducibility of your results. Protocols.io assigns your protocol its own identifier (DOI) so that it can be cited independently in the future. For instructions see:*

*https://journals.plos.org/plosone/s/submission-guidelines#loc-laboratory-protocols. Additionally, PLOS ONE offers an option for publishing peer-reviewed Lab Protocol articles, which describe protocols hosted on protocols.io. Read more information on sharing protocols at* [*https://plos.org/protocols?utm_medium=editorialemail&utm_source=authorletters&utm_campaign=protocols*](https://plos.org/protocols?utm_medium=editorialemail&utm_source=authorletters&utm_campaign=protocols)*.*

*We look forward to receiving your revised manuscript.*

*Kind regards,*

*Nicoletta Righini, PhD*

*Academic Editor*

*PLOS ONE*

*Journal Requirements:*

*Please review your reference list to ensure that it is complete and correct. If you have cited papers that have been retracted, please include the rationale for doing so in the manuscript text, or remove these references and replace them with relevant current references. Any changes to the reference list should be mentioned in the rebuttal letter that accompanies your revised manuscript. If you need to cite a retracted article, indicate the article’s retracted status in the References list and also include a citation and full reference for the retraction notice.*

*[Note: HTML markup is below. Please do not edit.]*

*Reviewers' comments:*

*Reviewer's Responses to Questions*

*Comments to the Author*

1. *If the authors have adequately addressed your comments raised in a previous round of review and you feel that this manuscript is now acceptable for publication, you may indicate that here to bypass the “Comments to the Author” section, enter your conflict of interest statement in the “Confidential to Editor” section, and submit your "Accept" recommendation.*

*Reviewer #1: (No Response)*

*2. Is the manuscript technically sound, and do the data support the conclusions?*

*The manuscript must describe a technically sound piece of scientific research with data that supports the conclusions. Experiments must have been conducted rigorously, with appropriate controls, replication, and sample sizes. The conclusions must be drawn appropriately based on the data presented.*

*Reviewer #1: Partly*

*3. Has the statistical analysis been performed appropriately and rigorously?*

*Reviewer #1: Yes*

*4. Have the authors made all data underlying the findings in their manuscript fully available?*

*The PLOS Data policy requires authors to make all data underlying the findings described in their manuscript fully available without restriction, with rare exception (please refer to the Data Availability Statement in the manuscript PDF file). The data should be provided as part of the manuscript or its supporting information, or deposited to a public repository. For example, in addition to summary statistics, the data points behind means, medians and variance measures should be available. If there are restrictions on publicly sharing data—e.g. participant privacy or use of data from a third party—those must be specified.*

*Reviewer #1: Yes*

*5. Is the manuscript presented in an intelligible fashion and written in standard English?*

*PLOS ONE does not copyedit accepted manuscripts, so the language in submitted articles must be clear, correct, and unambiguous. Any typographical or grammatical errors should be corrected at revision, so please note any specific errors here.*

*Reviewer #1: Yes*

*6. Review Comments to the Author*

*Please use the space provided to explain your answers to the questions above. You may also include additional comments for the author, including concerns about dual publication, research ethics, or publication ethics. (Please upload your review as an attachment if it exceeds 20,000 characters)*

*Reviewer #1: The revision does great job addressing my comments on the original submission. The methods and tables have been clarified, and the data points added to Figure 2 go a long way to improve the interpretation of the results. My concerns regarding conceptual limitations in the interpretation have been addressed in extensive changes to the introduction and discussion. I only have two comments that I think still need be addressed:*

1) *The introduction shouldn’t state that the study attempts to test predictions of local adaptation. The prediction in line 100 is currently speculative (“if we find a difference, this might be suggestive that there is adaptation”), but ultimately, adaptation can’t be inferred by looking only at phenotypic differences/similarities. Any phenotypic differences could instead represent neutral/maladaptive plasticity. At the same time, countergradient variation is local adaptation that results in phenotypic similarities between populations. The rest of the predictions laid out in the last few sentences of the introduction are appropriate.*

**We have followed reviewer’s suggestion and made a change in the introduction**

2) *The manuscript now appropriately states that it can’t parse genetic/environmental sources of phenotypic similarities/differences. However, this also means that the manuscript should be more speculative about what the results tell us about insect responses to climate change. I previously mentioned that if phenotypic similarities are due to a lack of evolution or plasticity, then lines 306 and 322 are correct in suggesting that some insect life history traits may be robust in the face of climate change. However, if the phenotypic similarities are due to countergradient variation, life history traits in these populations should instead show plasticity in response to climate change. I think these two statements should be removed (lines 306 and 322). The manuscript could just explain that future studies examining plasticity and evolution across populations are needed to predict responses to climate change.*

**We have followed reviewer’s suggestion and made those changes in the discussion**

7. PLOS authors have the option to publish the peer review history of their article (what does this mean?). If published, this will include your full peer review and any attached files.

If you choose “no”, your identity will remain anonymous but your review may still be made public.

Do you want your identity to be public for this peer review? For information about this choice, including consent withdrawal, please see our Privacy Policy.

Reviewer #1: No
